# Proxy Reports About Household Members With Increased Confusion or Memory Loss, 2011 Behavioral Risk Factor Surveillance System

**DOI:** 10.5888/pcd12.140427

**Published:** 2015-04-09

**Authors:** Valerie J. Edwards, Lynda A. Anderson, Angela J. Deokar

**Affiliations:** Author Affiliations: Lynda A. Anderson, Centers for Disease Control and Prevention, Atlanta, Georgia, and Emory University, School of Public Health, Atlanta, Georgia; Angela J. Deokar, Centers for Disease Control and Prevention, Atlanta, Georgia.

## Abstract

To provide information about the effects of increased confusion or memory loss (ICML) in households in the United States, we describe primary respondents’ reports (proxy reports) about another person in their household experiencing ICML, using 2011 Behavioral Risk Factor Surveillance System (BRFSS) data. We used proxy reports on type of assistance needed, effects on functioning in daily activities, and whether confusion or memory was discussed with a health care professional, stratifying by age of the household member with ICML (18–50 y vs ≥65 y). About 3% (n = 3,075 households) of primary respondents reported living with a household member with ICML; 75% of these household members needed some type of assistance, and nearly 60% had discussed ICML with a health care professional. Collecting proxy data about individuals in households may help paint a clearer picture of the characteristics of those experiencing cognitive decline and the potential needs of individuals and families.

## Objective

The Behavioral Risk Factor Surveillance System (BRFSS) has collected nationwide data on health risk behaviors and preventive practices associated with the leading causes of morbidity and mortality for more than 20 years (1). In 2011, information on increased confusion or memory loss (ICML) that occurred during the previous 12 months was collected for respondents, and if they themselves did not report ICML, they served as a proxy for a household member who was reported to be experiencing ICML. Because of initial concerns about self-disclosure of ICML, data were collected about other household members to provide a more comprehensive picture of ICML. The objective of this study was to describe the characteristics of US adults with ICML reported by proxy.

## Methods

Data were included from 13 states (Arkansas, Florida, Georgia, Hawaii, Illinois, Iowa, Louisiana, New Hampshire, North Carolina, South Carolina, Tennessee, West Virginia, and Wisconsin) that had available household weights and included ICML questions on their 2011 surveys (2). Primary respondents who were randomly selected via BRFSS protocol were asked the following screening question: “Have you experienced confusion or memory loss that is happening more often or is getting worse” (yes or no)? If more than 1 adult lived in the household, the primary respondent was also asked “How many adults in your household experienced confusion or memory loss that is happening more often or is getting worse during the past 12 months?” If the primary respondent answered no to the ICML screener (n = 81,012) but reported that someone else in the household experienced ICML, the primary respondent began serving as the proxy respondent and answered the 6 ICML questions about the household member with ICML (n = 3,075). When multiple household members experienced ICML, the proxy answered questions about the person with the most recent birthday. Age data for 30 household members with ICML were not available, and these records were excluded from further analyses; the final analytic sample (n = 3,045) was stratified by age (18–50 y vs ≥65 y) (Figure).

**Figure:**
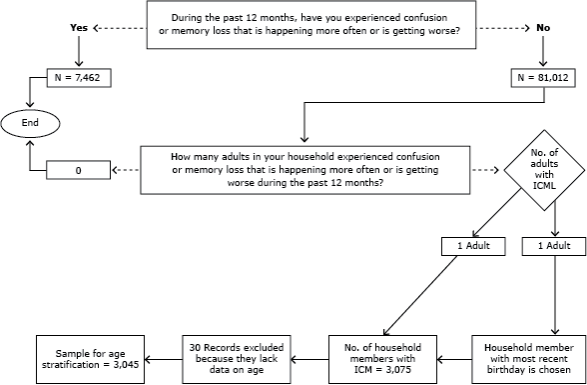
Classification of BRFSS respondents experiencing ICML themselves or serving as a proxy for another household member experiencing ICML, 2011. The questions on ICML were introduced with the following statement: “The next few questions ask about difficulties in thinking or remembering that can make a big difference in everyday activities. This does not refer to occasionally forgetting your keys or the name of someone you recently met, which is normal.” Abbreviations: BRFSS, Behavioral Risk Factor Surveillance System; ICML, increased confusion or memory loss.

Data were weighted using household weights based on the number of occupied households per state, derived from the American Community Survey Public Use Microdata Sample (PUMS) from the 2010 US Census (3). Household weights were used because household members are “nested” within households and the data do not conform to the random selection process used to compute individual-level weights.

Functional difficulties were assessed by asking the proxy whether ICML caused the household member to give up household activities or chores they used to do or interfered with their ability to work, volunteer, or engage in social activities (“always,” “usually,” or “sometimes” vs “rarely” or “never”). Additional questions asked about type of assistance most needed, if any, and if confusion or memory loss was discussed with a health care professional. Analyses were performed with SPSS version 22 (IBM Corp) to obtain weighted point estimates. Pairwise deletion was used for all analyses.

## Results

There were 3,075 proxies (weighted estimate, 3.7%; 95% confidence interval [CI], 3.5%–3.9%) who reported about a household member who experienced ICML. For the 3,045 household members with ICML for whom age information was available, about 74% were aged 60 years or older (60–69 y, 19.4%, 95% CI, 18.2%–20.6%; 70–79 y, 23.3%, 95% CI, 22.1%–24.6%; 80–89 y, 23.3%, 95% CI, 22.1%–24.6%; and aged ≥90 y, 8.1%, 95% CI, 7.4%–8.9%). About one-quarter were aged 18 to 59 (18–29 y, 2.0%, 95% CI, 1.8%–2.2%; 30–39 y, 3.8%, 95% CI, 3.4%–4.2%, 40–49 y, 7.5%, 95% CI, 6.8%–8.3%, and 50–59 y,12.6%, 95% CI, 11.7%–13.5%).

For household members aged 18 to 59, 68.5% (n = 463) were reported to need some type of assistance in 1 of 5 areas compared with about 76.5% (n = 1,711) of those aged 60 or older (Table). Household members aged 18 to 59 were reported to need the most assistance for household tasks; transportation was reported to require the most assistance for those aged 60 or older. In the area of functional difficulties, 51.9% of proxies reported that the household member had to give up household chores as a result of ICML, and 52.4% had given up work, volunteering, or social activities because of ICML, with the older adults having significantly more difficulties than younger adults. Finally, 59.0% of household members were reported by proxies to have discussed confusion or memory loss with a health care professional; significantly more of the older age group were reported to have had such discussions compared with the younger age group.

**Table:** Proxy Reports of Problems and Health Care Activity for Household Members With Reported ICML, 2011 Behavioral Risk Factor Surveillance System^a^

Type of Problem	Household Member Aged 18–59 y With ICML		Household Member Aged ≥60 y With ICML		All Proxy-Reported Household Members With ICML^b^ (n = 3,045)	
	Count	% (95% CI)	Count	% (95% CI)	Count	% (95% CI)
**Most assistance needed for . . .^c^**						
Safety	63	9.3 (7.9–10.9)	262	12.0 (11.1–1297)	325	11.3 (10.6–12.1)
Transportation	99	15.7 (14.1–17.6)	517	22.8 (21.3–24.4)	616	21.0 (19.8–22.)
Household activities	178	27.6 (25.1–30.2)	385	19.4 (17.9–20.9)	563	21.5 (20.2–22.8)
Personal care	90	12.3 (10.9–13.8)	454	19.2 (18.1–20.4)	544	17.5 (16.5–18.4)
Other area	33	3.6 (3.1–4.1)	93	3.1 (2.6–3.8)	126	3.2 (2.8–3.7)
None needed	206	31.5 (29.0–34.1)	524	23.5 (22.0–25.2)	730	25.6 (24.3–27.0)
**Functional difficulties**						
**Gave up household chores^d^**						
Never/rarely	361	52.0 (49.4–54.6)	1,043	46.7 (44.9–48.4)	1,404	48.1 (46.6–49.5)
Sometimes/usually/always	322	48.0 (45.4–50.6)	1,249	53.3 (51.6–55.1)	1,571	51.9 (50.5–53.4)
**Affected work and social life^e^**						
Never/rarely	375	58.4 (55.9–60.8)	1,114	50.3 (48.5–52.1)	1,489	52.4 (50.9–53.9)
Sometimes/usually/always	307	41.6 (39.2–44.1)	1,190	49.7 (47.9–51.5)	1,497	47.6 (46.1–49.1)
**Discussed confusion or memory loss with health professional^e^**	346	49.8 (47.2–52.4)	1,434	62.2 (60.5–63.9)	1,780	59.0 (57.6–60.4)

Abbreviation: CI, confidence interval; ICML, increased confusion or memory loss.

a Pairwise deletion used; therefore, totals are not identical for each item.

b Reports on 30 individuals were excluded from the age-stratified analysis.

c Proxy response to question “As a result of this person’s confusion or memory loss, in which area does this person need the *most* assistance?”

d *P* = .002.

e *P* < .001.

## Discussion

The BRFSS collected information from a primary respondent about another adult in a household for the first time in 2011. BRFSS data on children have been collected by randomly selecting a child in the household and obtaining information about that child from the primary respondent. This approach is a reliable way to obtain information on the health status of children who would not normally be included in BRFSS (4). In our study, data collected from proxies allowed us to obtain information about another household member experiencing ICML when the primary respondent did not report experiencing it. Our findings complement other recent findings on functional difficulties among people self-reporting ICML (5) and findings on ICML for entire households (2).

Household members whose ICML is reported by proxy respondents appear to be more impaired by ICML than are respondents who report their own ICML. For example, we found that nearly three-quarters of household members aged 60 or older were reported by proxies to need some type of assistance. In contrast, a recent study using BRFSS data reported that for primary respondents aged 60 or older, only about half reported needing assistance because of ICML (6). However, we note that the proxy data and the self-reported data are not directly comparable because of different weights. These findings also support the importance of examining information about the impact of diminished functioning among others in the household with reported ICML.

Limitations to these findings should be considered. Data on ICML are self-reported and are distinct from data on the prevalence of Alzheimer’s disease and related dementias. BRFSS respondents were drawn from households with a telephone landline, thereby limiting the population to those who can afford one and to those who do not live in institutionalized settings. Additionally, data cannot be construed as a national average. Furthermore, research indicates selective bias by age in over- and underreporting disabilities for others (7). Collecting data from proxies about household members may help paint a clearer picture about the characteristics of those experiencing cognitive decline and the potential burden faced by individuals and their families.
